# OH defect contents in quartz in a granitic system at 1–5 kbar

**DOI:** 10.1007/s00410-019-1632-0

**Published:** 2019-11-11

**Authors:** Alexander Potrafke, Roland Stalder, Burkhard C. Schmidt, Thomas Ludwig

**Affiliations:** 1 Institut für Mineralogie und Petrographie, Universität Innsbruck, Innrain 52f, 6020 Innsbruck, Austria; 2 Abteilung für Experimentelle und Angewandte Mineralogie, Geowissenschaftliches Zentrum, Georg-August Universität Göttingen, Goldschmidtstraße 1, 37077 Göttingen, Germany; 3 Institut für Geowissenschaften, Universität Heidelberg, Im Neuenheimer Feld 234-236, 69120 Heidelberg, Germany

**Keywords:** Quartz, OH defects, IR spectroscopy, Granite

## Abstract

Quartz is able to incorporate trace elements (e.g., H, Li, Al, B) depending on the formation conditions (P, T, and chemical system). Consequently, quartz can be used as a tracer for petrogenetic information of silicic plutonic bodies. In this experimental study, we provide the first data set on the OH defect incorporation in quartz from granites over a pressure/temperature range realistic for the emplacement of granitic melts in the upper crust. Piston cylinder and internally heated pressure vessel synthesis experiments were performed in a water-saturated granitic system at 1–5 kbar and 700–950 °C. Crystals from successful runs were analysed by secondary ion mass spectrometry (SIMS) and Fourier transform infrared (FTIR) spectroscopy, and their homogeneity was verified by FTIR imaging. IR absorption bands can be assigned to specific OH defects and analysed qualitatively and quantitatively and reveal that (1) the AlOH band triplet at 3310, 3378 and 3430 cm^−1^ is the dominating absorption feature in all spectra, (2) no simple trend of total OH defect incorporation with pressure can be observed, (3) the LiOH defect band at 3470–3480 cm^−1^ increases strongly in a narrow pressure interval from 4 kbar (220 µg/g H_2_O) to 4.5 kbar (500 µg/g H_2_O), and declines equally strong towards 5 kbar (180 µg/g H_2_O). Proton incorporation is charge balanced according to the equation H^+^ + A^+^ + P^5+^ = M^3+^ + B^3+^, with A^+^ = alkali ions and M^3+^ = trivalent metal ions.

## Introduction

Quartz is the second most abundant mineral in the Earth’s crust and an important rock-forming constituent in igneous, metamorphic and sedimentary rocks as well as unconsolidated sedimentary material (Ronov and Yaroshevski 1969). Quartz does not form solid solution series, but is capable of incorporating considerable amounts of trace elements such as P^5+^, Ti^4+^, Ge^4+^, Al^3+^, Fe^3+^, B^3+^, Li^+^, Na^+^, K^+^ and H^+^ (Bambauer 1961, 1963; Kats 1962; Aines and Rossman 1984; Müller and Koch-Müller 2009), following the general charge balance equation H^+^ + A^+^ + P^5+^ = M^3+^ + B^3+^, with A^+^ = concentrations (a.p.f.u.) of alkali ions and M^3+^ = concentrations (a.p.f.u.) of trivalent metal ions (Müller and Koch-Müller 2009; Frigo et al. 2016). Trace protons form hydroxyl groups (OH^−^) with the oxygen anions from the quartz lattice and, since oxygen is the only relevant anion, its concentration can be expressed as neutral water component. FTIR-spectroscopy is a powerful tool to investigate hydrous defects in nominally anhydrous minerals qualitatively (OH defect species) and quantitatively (OH defect content). IR spectra give rise to absorption bands at specific wavenumbers that can be assigned to particular hydrous defects: (1) the substitution Si^4+^ = Al^3+^ + H^+^ (referred to as AlOH defect) is thermally rather stable and is responsible for the band triplet at 3310, 3378 and 3430 cm^−1^ (Kats 1962; Bambauer 1963; Aines and Rossman 1984; Rovetta 1989; Stalder and Konzett 2012), (2) the interstitial LiOH defect leads to an absorption band in the range of 3470–3482 cm^−1^ (Kats 1962; Aines and Rossman 1984; Baron et al. 2015), while (3) the absorption band at 3595 cm^−1^ is related to the substitution Si^4+^ = B^3+^ + H^+^ and called BOH defect (Müller and Koch-Müller 2009), usually associated with late-stage granitic to pegmatitic origin. Furthermore, a subordinate band at 3585 cm^−1^ is assigned to a defect analogous to the hydrogarnet substitution (Si^4+^ = 4H^+^), which has been identified almost exclusively in synthetic quartz from high-pressure experiments (Rovetta 1989; Stalder and Konzett 2012) and is practically insignificant in natural quartz from the Earth’s crust (Stalder 2014).

OH defect incorporation, as trace element incorporation in general, is controlled by physico-chemical parameters such as pressure, temperature, chemical composition and water fugacity. The incorporation of trace metals has successfully been applied as geothermometer (Wark and Watson 2006; Huang and Audétat 2012). Similarly, OH defects in quartz provide detailed information on the prevailing conditions in granitic melts and thus are ideal tracers for crystallisation conditions. In order to better understand the influence of the different parameters controlling OH defect incorporation, experiments under defined conditions are able to imitate natural processes and—ultimately—help to decipher findings made in natural rocks. In this context, recent experimental studies on OH defect incorporation in quartz revealed (1) a negative pressure dependence of OH defect content with increasing pressure in the range of 5–20 kbar (Baron et al. 2015; Frigo et al. 2016), (2) a decrease of LiOH defect content with increasing pressure, until at > 10 kbar LiOH is absent (Frigo et al. 2016), and (3) an increase of hydrogarnet defects with increasing pressure and water activity (Stalder and Konzett 2012).

Although a broad overview of hydrous defects in quartz from natural granitic starting materials over crustal conditions has been established, the most relevant pressure range for granite solidification (< 5 kbar corresponding to < 15 km depth) has not been studied in detail. In this study, we present the first systematic experimental data set on the OH defect incorporation in quartz from a natural granitic starting material at 1–5 kbar, representing relevant conditions of granite genesis in upper to mid crustal levels. Our findings will be discussed as tool to decipher differentiation trends in natural granitic bodies.

## Experimental and analytical methods

### Starting material

Quartz crystals were synthesised from a natural granitic starting material artificially enriched in quartz and water. For better comparability we used the same natural granite from Sardinia/Italy as Frigo et al. (2016) with a composition of 68.9 wt% SiO_2_, 14.9 wt% Al_2_O_3_, 6.35 wt% K_2_O, 3.05 wt% Na_2_O, 2.51 wt% Fe_2_O_3_, 1.56 wt% CaO, 0.50 wt% MgO, 0.27 wt% TiO_2_, 0.08 wt% MnO, 0.04 wt% P_2_O_5_, doped with additional synthetic quartz (Alfa Aesar 99.995% purity). The artificial enrichment of quartz in the starting material was necessary to promote the growth of sufficiently large crystals, and to ensure that quartz is the primary phase on the liquidus. Both components were ground to powder in an agate ball mill for 1 h, mixed in proportion of 1:1 and milled again for 1 h for homogenisation.

### Experimental strategy

**Fig. 1:**
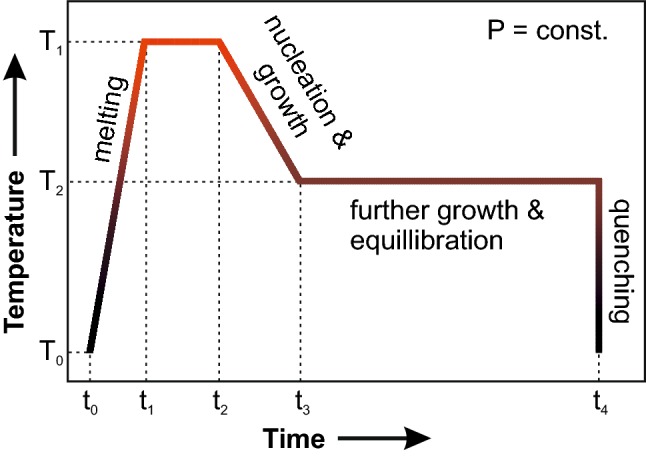
Temperature–time profile of the experimental strategy. The temperature was raised significantly above the estimated liquidus temperature and kept there for 24 h (T_1_), followed by successive cooling along a temperature ramp between 6 and 18 °C/h, and equilibrated at the target temperature (T_2_) at constant pressure for 24–157 h before quenching

Melts with high silica contents are strongly polymerised and exhibit high viscosities and low mobility of chemical components. Yet, water reduces the viscosity significantly, but at low pressure the water solubility in melts is comparatively low and the effect on element mobility is limited. Thus, crystal growth is very sluggish and synthesis of sufficiently large crystals in reasonable time is challenging. Consequently, quartz crystals usually stay small (< 10 µm) and become cemented in the quench glass, and cannot be used for polarised IR measurements. In order to improve the synthesis conditions, a set of pre-experiments with variable run parameters (water content, degree of overheating above liquidus, degree of undercooling below liquidus, temperature ramp) was conducted to develop an experimental strategy that enhances crystal growth and suppresses nucleation. Additionally, temperature cycling after the method of Erdmann and Koepke (2016) was tested in two experiments (cycling with ± 20 °C around a given temperature for 40 min each) but was not further followed for the final strategy since the effect was negligible. The most successful experimental setting was (1) heating to a temperature significantly above the estimated liquidus followed by a short dwell, (2) cooling along a ramp towards the end temperature with a cooling rate between 1 and 100 °C/h, and (3) holding the end temperature for at least 24 h to equilibrate the synthesised quartz crystals (Fig. 1). The largest crystals (> 150 μm) were produced when starting and final temperature differed by more than 200 °C. These crystals grew to 100% from the melt, thus they have no relict core and no inherited OH defects.

### High pressure experiments

The powdered starting material was filled in either Pt or Au capsules with outer (inner) diameter of 4.0 (3.6) mm or 3.0 (2.6) mm, depending on run conditions and experimental assembly (Pt: > 1000 °C, Au: < 1000 °C). 6–18 wt% distilled water was added to ensure water saturation. After filling the capsule was welded, weighted, heated for at least 1 h in an oven at 125 °C, and weighted again in order to check for leakage.

Experiments at 4–5 kbar were conducted using either a Johannes Type or an end-loaded Boyd-England Type piston cylinder (PC) at the University of Innsbruck (Table 1). Capsules were placed vertically into an MgO-sleeve, which was inserted into a 22 mm NaCl-Pyrex assembly with graphite furnace. The pressure was calibrated using the water in albite-glass geobarometer (Baker 2004). The temperature was monitored using a Ni–CrNi thermocouple and all runs were quenched by switching off the power supply.

**Table 1:** Experimental conditions and starting material

Run#	Press type	Capsule (mm)	Granite (mg)	Quartz (mg)	Water (mg)	P (kbar)	T (°C)	Ramp (°C/h)	Dwell (h)
IHPV23	IHPV	Pt 4.0	59.6	59.6	6.4	1.0	1200–800	10	104
IHPV21a	IHPV	Pt 4.0	80.4	80.4	19.8	2.0	1200–800	10	64
IHPV21b	IHPV	Pt 4.0	69.8	69.8	24.0	2.0	1200–800	10	64
IHPV03	IHPV	Pt 4.0	28.6	28.6	10.6	3.0	1200–950	> 200	24
PC01	PC “J”	Pt 3.0	23.8	23.8	7.0	4.0	900–700	6	35
PC06	PC “BE”	Pt 4.0	25.9	25.9	7.9	4.5	900–700	6	157
PC09	PC “BE”	Pt 4.0	25.1	25.1	7.2	5.0	900–700	10	54

*IHPV* internally heated pressure vessel, *PC* piston cylinder, *J* Johannes, *BE* Boyd and England

Experiments at 1–3 kbar were performed in an internally heated pressure vessel (IHPV) with argon gas as pressure medium at the University of Göttingen, Germany. The oven volume of the IHPV is large enough to store up to five capsules, enabling the contemporaneous run of different starting materials at the very same conditions. A more detailed description of the vessel and operation conditions is given in Schmidt and Behrens (2008). The temperature was measured by three Pt-PtRh10 thermocouples and computer-controlled during the entire run duration. All runs were quenched isobarically by switching off the power supply and maintaining the pressure in the vessel with the help of a pressure intensifier.

Recovered capsules were weighed, pierced, dried and weighed again to exclude fluid loss during the experimental run, and the experimental charge was inspected under an optical microscope. Escape of water during piercing and bubbles in the glassy portion of the run product were regarded as proof for water-saturated conditions. The excess fluid was investigated by pH paper, exhibiting neutral to very weak acidic values (pH 6.5–7) for all runs.

### Sample preparation

Depending on the amount of sufficiently large grains in the run product, 4–12 oriented quartz crystals per experimental charge were prepared for IR-spectroscopy following the protocol described by Stalder and Konzett (2012). Quartz crystals were individually handpicked from the run product and embedded in thermoplastic resin on a glass slide. The crystal alignment was checked with an optical microscope. Successful orientation was confirmed by birefringence values of Δ*n* = 0.009 in orthoscopic and “flash figures” in conoscopic illumination. Oriented crystals were manually polished on both sides and remaining resin was removed by rinsing in acetone. The crystal thickness, a crucial parameter for water quantification, was determined by a mechanical Mitutoyo micrometer with an accuracy of ± 2 μm, and crosschecked by the absorption band of the lattice overtone at 1793 cm^−1^ (Stalder et al. 2017).

**Fig. 2:**
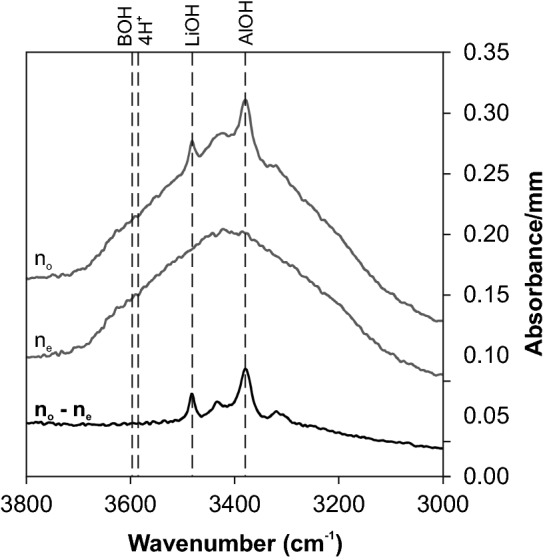
Example of IR spectra treatment for OH determination. The molecular water signal is eliminated by subtracting the isotropic portion (E||n_e_) from the polarised portion (E||n_o_) caused by OH defects. Spectra are offset vertically for graphical reasons

In contrast to most other methods, polarised IR-spectroscopy measurements are able to distinguish between molecular water, causing a broad band between 3000 and 3700 cm^−1^, and water from OH defects, causing rather sharp absorption bands at characteristic wavenumbers between 3250 and 3600 cm^−1^. Most OH defect absorption bands are perfectly polarised perpendicular to the optical axis (E||n_o_), whereas the molecular water signal exhibits isotropic behaviour (identical absorbance in all crystallographic directions). Thus, the polarised measurement E||n_o_ reveals the OH defects plus molecular water, E||n_e_ reveals the molecular water only, and the contribution of the OH defects is derived by the subtraction E||n_o_–E||n_e_ (Fig. 2). The only deviation from this protocol is the B-related OH absorption band at 3595 cm^−1^ that is not perfectly aligned perpendicular to the optical axis and shows a small contribution for E||n_e_ (Baron et al. 2015). However, this band was considered negligible for crystals from this study and no correction was performed.

### FTIR spectroscopy and OH defect quantification

Near- to Mid-infrared spectra were recorded at room temperature in transmission mode by using a Bruker Vertex 70 FTIR spectrometer coupled to a Hyperion 3000 microscope, nitrogen-cooled MCTD316-025 (mercury cadmium telluride) detector, a SiC globar light source, a KBr beamsplitter and a wire ZnSe grid polariser. The beam path was continuously flushed with dry air and samples were placed on a BaF_2_ plate. 50–500 scans were conducted on background and sample with a resolution of 4 cm^−1^ and spot sizes between 40 × 40 and 120 × 120 μm in the wavenumber range of 7000–550 cm^−1^. On each crystal one to three measurements were performed on virtually inclusion-free spots.

The two polarised IR spectra from each spot were subtracted (E||n_o_–E||n_e_), normalised to thickness and baseline-corrected by a linear baseline between 3600 and 3250 cm^−1^. OH defect concentrations (Table 2) were calculated by integration in the spectral region (3600–3250 cm^−1^), using the integrated extinction coefficients from mineral-specific (Aines et al. 1984; Thomas et al. 2009) and general wavelength-specific (Libowitzky and Rossman 1997) calibrations. Results are expressed as µg/g H_2_O, which is equivalent to the notation ppm H_2_O.

**Table 2:** Prepared crystals and OH defect content. Errors are ± 10% for OH defect content and ± 2 μm for thickness determination

Crystal#	P (kbar)	Thickness (µm)	A	OH-defect content (µg/g)		
				T (09)	L&R (97)	A (84)
IHPV23						
qz1	1.0	57	124.2	189.6	187.7	195.6
qz2	1.0	50	140.9	215.5	208.5	221.9
qz3	1.0	46	143.0	218.3	207.4	225.3
qz4	1.0	48	131.2	200.3	209.1	206.6
IHPV21a						
qz1	2.0	70	89.9	137.2	136.0	141.6
qz2	2.0	69	96.0	146.5	144.9	151.2
qz3	2.0	65	103.5	157.9	155.4	163.0
qz4	2.0	75	88.3	134.8	132.9	139.1
qz5	2.0	73	96.9	147.9	144.7	152.6
IHPV21b						
qz1	2.0	90	101.3	154.6	150.5	159.5
qz2	2.0	80	92.9	141.7	140.3	146.3
IHPV03						
qz1	3.0	82	111.1	169.5	166.5	174.9
qz2	3.0	80	124.5	190.0	185.7	196.0
qz3	3.0	78	113.6	173.4	170.0	178.9
qz4	3.0	46	93.8	143.1	142.4	147.7
qz5	3.0	57	124.7	190.3	189.0	196.3
PC01						
qz1	4.0	72	134.5	205.3	206.0	211.8
qz2	4.0	77	131.7	201.0	199.1	207.4
qz3	4.0	70	166.4	254.0	252.9	262.1
qz4	4.0	105	117.4	179.1	177.6	184.8
qz5	4.0	86	252.2	385.0	384.8	397.2
qz6	4.0	87	111.1	169.5	169.6	175.0
qz7	4.0	106	198.8	303.4	303.1	313.1
qz8	4.0	82	179.9	274.6	274.1	281.1
qz9	4.0	74	226.2	345.3	344.6	356.3
qz10	4.0	76	106.9	163.2	162.9	168.4
qz11	4.0	63	222.5	340.0	341.5	350.5
qz12	4.0	77	107.4	164.0	164.1	169.2
PC06						
qz1	4.5	75	310.0	474.3	532.4	489.4
qz2	4.5	60	354.1	540.5	602.8	557.7
qz3	4.5	68	217.5	332.1	356.8	342.6
qz4	4.5	75	290.8	443.9	491.7	458.0
PC09						
qz1	5.0	83	75.7	115.5	115.4	119.2
qz2	5.0	85	141.4	215.8	214.5	222.7

*A* integrated absorbances in the range of 3250–3600 cm^−1^

*T (09)* according to the calibration of Thomas et al. (2009)

*L&R (97)* according to the calibration of Libowitzky and Rossman (1997)

*A (84)* according to the calibration of Aines et al. (1984)

### IR Imaging

In order to visualise the chemical homogeneity, the OH distribution of selected crystals was also monitored using the focal plane array (FPA) detector of the Hyperion 3000 IR microscope. The FPA consists of 64 × 64 MCTD364 detectors, resulting in 4096 simultaneously recorded spectra in the range of 4000–900 cm^−1^ on an area of 170 × 170 µm. This implies that the spatial resolution of the MIR-signal is limited by the wavelength of the IR-radiation (e.g. 3 µm at 3300 cm^−1^). A larger area can be imaged by sequential analysis of several IR images. All spectra were automatically integrated in different spectral regions of interest, thus enabling a quick overview over the OH absorption bands, the absorptions caused by the lattice overtones (calculating the thickness by the method of Stalder et al. 2017), and the ratio of both for automatic thickness normalisation.

### Secondary ion mass spectrometry (SIMS)

Concentrations of Li, B, Na, Al, K, Ti and Ge were measured using a CAMECA ims3f ion microprobe at the Institute of Earth Sciences, Heidelberg University. ^16^O^−^ primary ions with a net energy of 17 keV and a beam current of ~ 15 nA were focused to a spot diameter of ~ 15 µm on the sample. Positive secondary ions were accelerated to 4.5 keV and the energy acceptance of the double-focussing mass spectrometer was set to 75 ± 20 eV (energy filtering). The mass resolving power M/ΔM was ~ 2000 (10%) and the secondary ions were detected by an electron multiplier in counting mode. To reduce the influence of surface contamination the area analysed was limited to a diameter of ~ 12 µm using a 750 µm field aperture and a nominal imaged field of 25 µm (Marschall and Ludwig 2004). Li, B, Na, Al, K, Ti and Ge concentrations in the unknown samples were calculated using the ion yield relative to Si (RIY) which was determined using the NIST SRM610 glass (concentrations taken from Jochum et al. 2011). The most critical isotope for molecular interference in this setup was ^74^Ge with these interferences: ^58^Fe^16^O, ^28^Si^30^Si^16^O and ^29^Si_2_^16^O. The FeO interference was negligible in the SRM610 glass and in the quartz samples, while the contribution of the ^28^Si^30^Si^16^O and ^29^Si_2_^16^O interference on the ^74^Ge peak was reduced to ≤ 0.1% of its intensity by mass resolving power (M/ΔM ~ 1500 at 0.1%). The influence of the ^28^Si^30^Si^16^O and ^29^Si_2_^16^O interference on ^74^Ge was also tested on the Herasil 102 quartz glass (Table 3). SIMS measurements were conducted on two to four crystals per experiment depending on the number of crystals suitable for analysis. On each crystal, two to five spots were measured depending on the crystal size. Data points with elevated concentrations of K and Al (K > 100 µg/g, Al > 5000 µg/g) were considered as contaminated by micro inclusions (fluid, melt or mineral) and hence rejected.

**Table 3:** Average trace element content from SIMS analyses

	Li (µg/g)	B (µg/g)	Na (µg/g)	Al (µg/g)	K (µg/g)	Ti (µg/g)	Ge (µg/g)
IHPV21a + b	2.1 (± 0.2)	0.07 (± 0.02)	3.0 (± 0.3)	373 (± 2)	4.4 (± 0.8)	149.6 (± 0.9)	1.9 (± 0.3)
IHPV03	1.6 (± 0.1)	0.04 (± 0.01)	1.0 (± 0.1)	336 (± 1)	1.3 (± 0.3)	132.9 (± 0.9)	1.2 (± 0.3)
PC01	6.3 (± 0.4)	0.05 (± 0.01)	6.2 (± 0.4)	606 (± 2)	1.7 (± 0.3)	224.9 (± 0.9)	0.4 (± 0.1)
PC06	83.7 (± 2.1)	0.19 (± 0.03)	73.4 (± 2.5)	2242 (± 13)	49.2 (± 0.9)	98.7 (± 0.6)	0.2 (± 0.1)
PC09	5.8 (± 0.4)	0.07 (± 0.02)	3.6 (± 0.2)	449 (± 2)	3.1 (± 0.6)	246.2 (± 1.2)	0.3 (± 0.1)
Herasil 102_1_	0.08 (± 0.01)	0.02 (± 0.01)	0.19 (± 0.06)	8.14 (± 0.67)	0.33 (± 0.08)	0.05 (± 0.02)	0.26 (± 0.22)

_1_silica glass

## Results

### Experimental run products

**Fig. 3:**
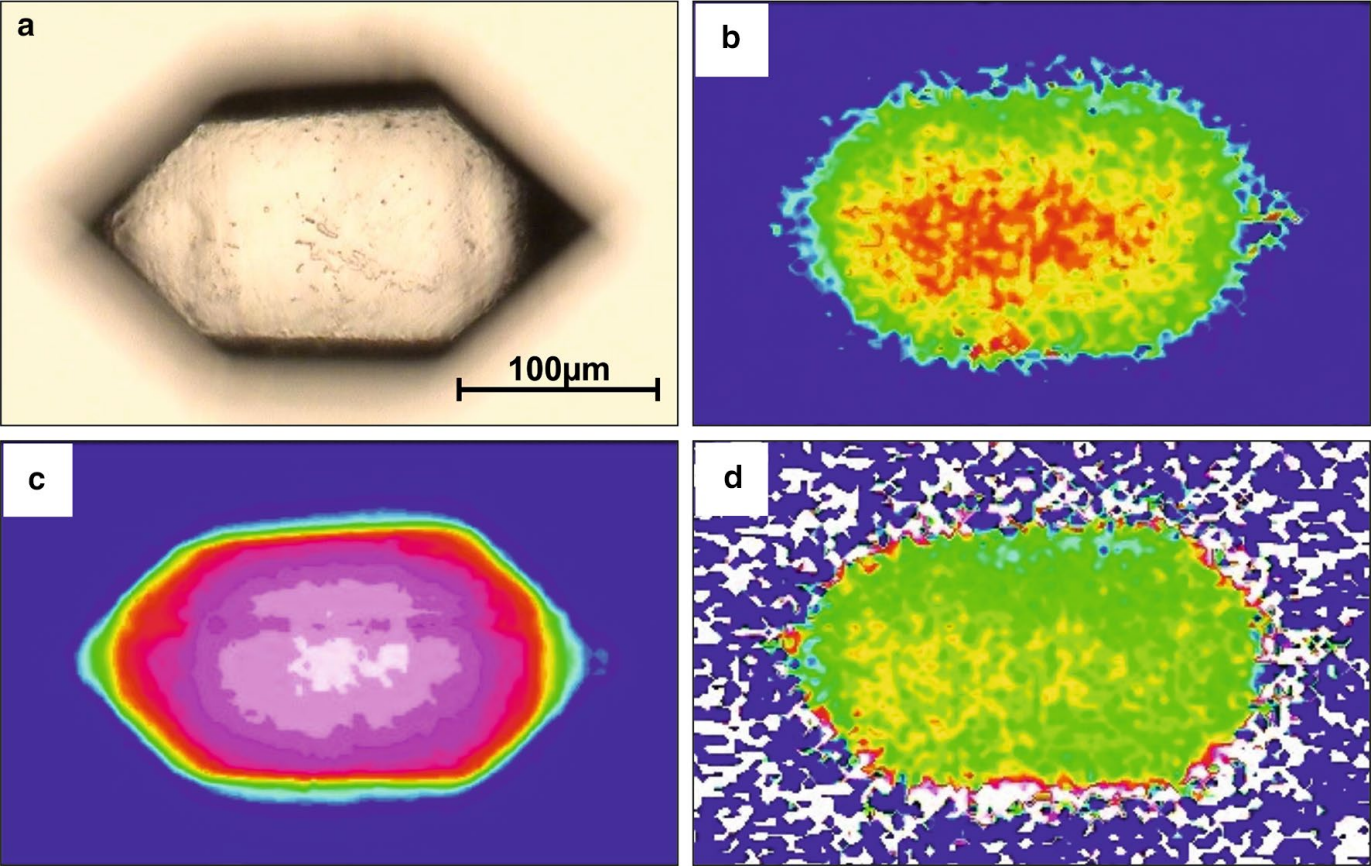
Euhedral quartz crystal from run PC01 at 4 kbar: **a** optical image, **b** IR image of OH distribution, integration range 3200–3650 cm^−1^, **c** thickness revealed by lattice overtones, integration range 1750–2100 cm^−1^, **d** OH distribution normalised to thickness. Warmer colours represent higher values

Run products from most experiments mainly contained quartz and glass. Furthermore, K-feldspar, phengite and/or magnetite were observed as accessory phases. Depending on the supersaturation of H_2_O in the starting material and thus on the amount of gas bubbles, the texture of the recovered material ranged from solid glass blocks to fragments, from which euhedral quartz crystals > 100 µm (Fig. 3) could directly be handpicked. Sporadically, arrays of small crystals with identical optical orientation (probably sections of the same skeletal crystal) were observed. Crystal sizes and habits strongly depend on the water content of the starting material, start and end temperature, and the cooling rate.

### OH defect content and spatial distribution

**Fig. 4:**
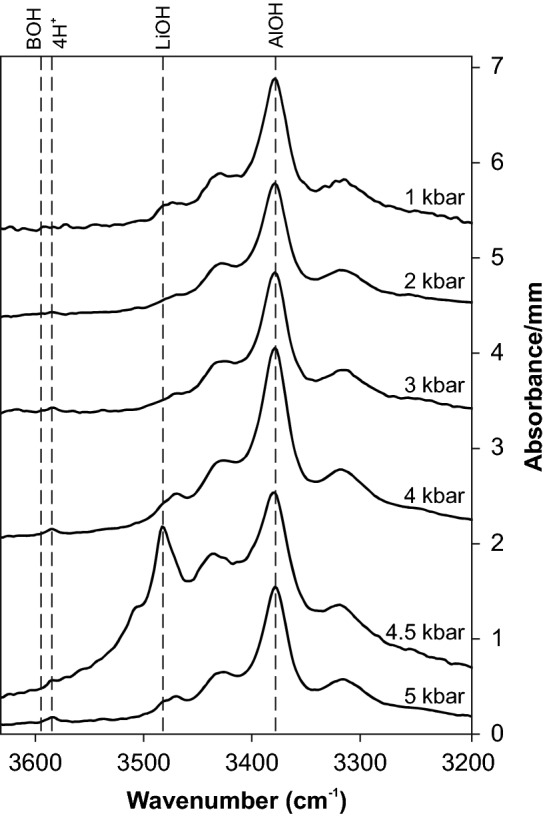
Average IR spectra (E||n_o_-E||n_e_) at different pressures. Spectra are normalised to 1 mm thickness and offset vertically for graphical reasons

**Fig. 5:**
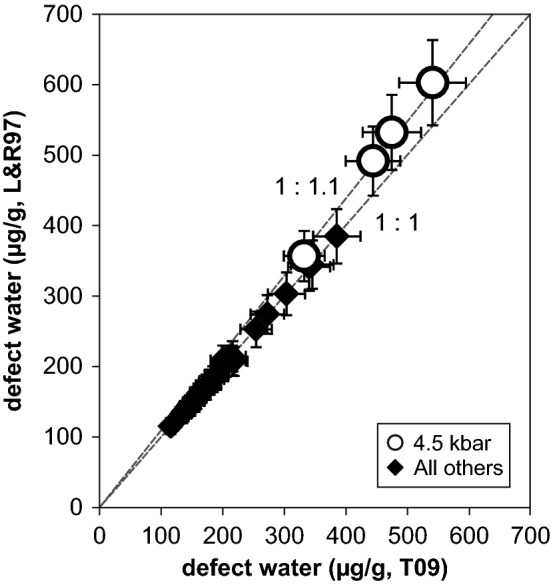
Comparison of total OH defect contents (expressed as water equivalent) calculated by the wavelength-specific calibration (L&R97) of Libowitzky and Rossman (1997) and the mineral specific calibration (T09) of Thomas et al. (2009). Results plot on the 1:1 line within error; slight, but significant deviations leading to higher values for the calibration L&R97 for crystals with increased LiOH absorption bands (open circles) because of the lower average extinction coefficient

In the MIR OH stretching region all spectra are dominated by the Al-specific OH triplet at 3310, 3378 and 3430 cm^−1^ (Fig. 4), with minor fluctuation throughout the investigated pressure range. The Li-specific defect at 3470–3480 cm^−1^ is subordinate in crystals from all experiments except run PC06 at 4.5 kbar, where the Li-specific absorption band shows strong contribution to the IR spectrum. This is expressed by an increase of the average LiOH/AlOH ratio to 0.9 (4.5 kbar) from 0.15 (all others). The hydrogarnet defect (Si^4+^ = 4H^+^) at 3585 cm^−1^ is discernible but negligible, while the BOH defect is absent throughout all experiments. Defect water contents range between 100 and 600 µg/g water (Table 2). All three used calibrations yield results in very good agreement with each other. Crystals with significant contributions of absorption bands at high wavenumbers (> 3400 cm^−1^) in the IR spectra deviate systematically from the 1:1 line towards higher values (Fig. 5) if the wavenumber-specific calibration (Libowitzky and Rossman 1997) is applied. This is logical because the average extinction coefficient (*ε*) is systematically—though slightly—lower, with *ε* = 67,000 ${\text{l mol}}_{{{\text{H}}_{2} {\text{O}}}}^{ - 1} {\text{cm}}^{ - 2}$ for the LiOH band position at 3480 cm^−1^ and *ε* = 92,000 ${\text{l mol}}_{{{\text{H}}_{2} {\text{O}}}}^{ - 1} {\text{cm}}^{ - 2}$ for the AlOH band position at 3380 cm^−1^, e.g., compared to the mineral specific calibration (Thomas et al. 2009) with a uniform *ε* = 89,000 ${\text{l mol}}_{{{\text{H}}_{2} {\text{O}}}}^{ - 1} {\text{cm}}^{ - 2}$. The analytical error for water quantification is estimated at 10%. Error sources are (1) mismatch in crystal orientation, (2) uncertainties in thickness determination, and (3) error in baseline correction.

IR imaging reveals a significant increase in both OH absorptions and lattice overtones towards the centre of the crystal (Fig. 3b, c), mostly reflecting the slightly biconvex shape of the crystal that developed during manual preparation. OH absorptions normalised to thickness show a very weak zonation (Fig. 3d) suggesting more or less homogeneous OH defect distribution.

### Trace element contents

**Fig. 6:**
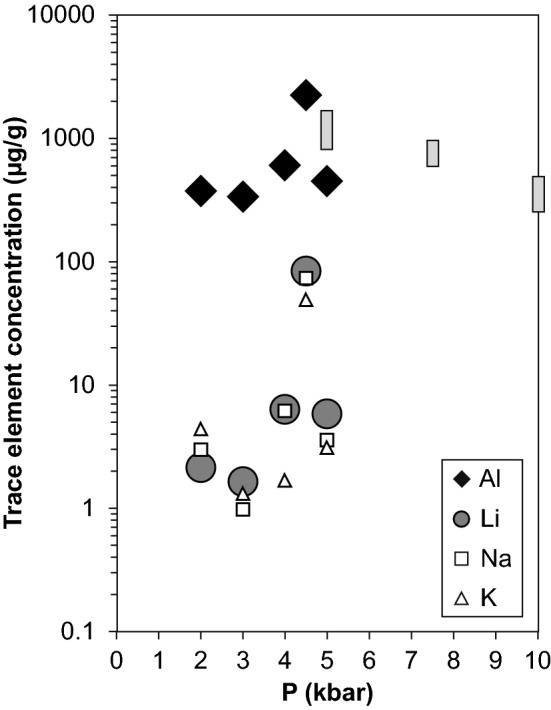
Trace element concentrations in quartz from SIMS analyses for Al, Li, Na and K against pressure. Grey areas represent the results of Frigo et al. (2016) for Al. Errors (this study) are not indicated because they are smaller than the size of the symbol

In all analysed grains, Al is the most abundant trace element and exhibits very low variations within one experimental charge. All other trace metals that can serve as charge balance to incorporate protons (Li, B, Na, K) are in the lower µg/g range in most runs (Table 3). The only exception is run PC06 (4.5 kbar), where Li, Na and K are enriched by more than an order of magnitude, and Al is enhanced by a factor of 5 (Fig. 6). Especially Li becomes important for charge balance in run PC06, which is in accordance with the IR spectra, where the LiOH band is strongly enhanced and nearly as prominent as the AlOH absorption band. The tetravalent elements Ti and Ge do not seem to be affected by pressure. Titanium, however, does not provide reliable results for geothermometry (Wark and Watson 2006; Huang and Audétat 2012), since rutile was absent in all experiments.

## Discussion

### Attainment of equilibrium

The observation of crystal habits of different stages between skeletal and euhedral illustrates the evolution of crystal growth (Mollard et al. 2012). Skeletal crystals represent the early stage of crystal growth, while euhedral crystals are interpreted as the final stage. Euhedral crystals, in turn, probably all went through the skeletal stage and therefore may contain chemical information that was inherited from an earlier stage of crystallisation (higher temperatures). The homogeneous OH distribution (Fig. 3d) in combination with the euhedral crystal habits is interpreted as evidence for sufficiently long annealing at final run temperatures.

### Crystal chemistry

**Fig. 7:**
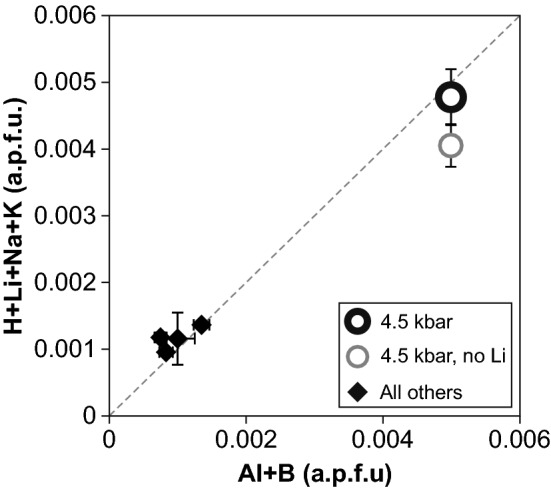
Sum of monovalent against trivalent cations (averaged over each experiment) expressed as atoms per formula unit (a.p.f.u.). The dashed line represents the charge balancing equation suggested by Müller and Koch-Müller (2009), excluding phosphorus. If not indicated, errors are within the size of the symbol

Impurity contents of Al, Li and total defect water show a maximum concentration at 4.5 kbar and fit precisely to the proposed charge balancing equation H^+^ + A^+^ + P^5+^ = M^3+^ + B^3+^, with A^+^ = alkali ions and M^3+^ = metal ions (Müller and Koch-Müller 2009). The data also show the contrasting role of Li^+^—namely forming a dry AlLi defect versus the formation of a hydrous LiOH defect associated with non-bridging oxygens—and underline that Li^+^ competes against H^+^ for charge balancing the trivalent cations (Fig. 7). Phosphor, however, has not been included in the SIMS protocol for the reason that analyses of P require extensive modifications in the measurement strategy at the expense of the detection limits of other trace elements. Since the data already fit to the 1:1 line and because constant low P contents have been detected in synthetic quartz from experiments in the adjacent pressure range (5–20 kbar) and the same starting material (Frigo et al. 2016), it can be safely assumed that P is a minor component and can be neglected in the charge balancing equation in this particular study.

### Defect water contents

**Fig. 8:**
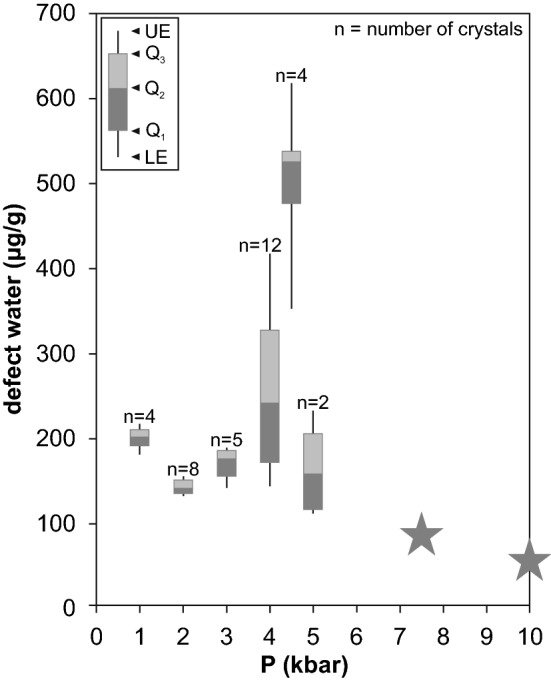
Total OH defect contents (expressed as water equivalent) against pressure. Data points at 7.5 and 10 kbar (asterisk) are derived from Frigo et al. (2016) for comparison. *UE* upper extreme, *LE* lower extreme, *Q*_*1*_ 25th percentile, *Q*_*2*_ median, *Q*_*3*_ 75th percentile

Aluminum is the most abundant trace element in quartz and hence the Al-specific OH species dominate the hydrous defect inventory with only minor variation over the whole pressure range (Fig. 8). This monotony is interrupted between 4 and 4.5 kbar by a rapid increase of the LiOH defect and an equally strong descent towards 5 kbar. The increase of the LiOH band results in a change in LiOH/AlOH ratio from 0.15 at 4 kbar to nearly unity at 4.5 kbar, accompanied by a shift of the respective absorption band from 3470 to 3482 cm^−1^. The shift of the band position may be caused by a structural distortion of the defect at higher concentrations or might represent a further absorption band (Kats 1962).

The overall OH content does not show a distinct trend within the examined pressure range with rather constant values around 180–200 µg/g, except for the rapid increase at 4.5 kbar. These values are in the same range as previously published data on OH defect content in synthetic quartz (Baron et al. 2015; Frigo et al. 2016) but exceed those of average natural quartz from variscan granites (20 μg/g, Stalder et al. 2017) and the crustal average (10 μg/g, Stalder 2014) by an order of magnitude. This is most likely attributed to slow cooling of granitic bodies after solidification and hence long residence times at elevated temperatures, during which hydrogen partially diffuses out of the crystal structure. This also happens during metamorphism (very low to low grade) in the geological history of plutonic bodies. On the other hand, very high OH contents (> 100 µg/g) have occasionally been reported in quartz grains from siliciclastic material (Stalder 2014; Jaeger et al. 2019) and observed in natural pegmatite samples (unpublished data). These grains probably represent quartz crystals grown under water-saturated conditions that were not changed by later dehydrogenation. It should also be underlined that the experiments were conducted water-saturated and the fluid compositions have high water activities (dilution comes mainly from silica dissolution in the fluid). This might differ from natural granite systems, where H_2_O saturation is not necessarily given and fluids are most likely multi-component (aH_2_O≪1).

In combination with earlier studies that revealed a negative trend in the adjacent pressure range from 5–25 kbar (Baron et al. 2015; Frigo et al. 2016), the new data indicate a narrow interval of highly favourable pressure conditions for OH defect incorporation in quartz at 4–5 kbar. An artificial origin of the rapid increase at 4.5 kbar can be excluded, since no changes in starting material, sample assembly and experimental setup were made, and the monitored values of the pressure and temperature control document an error-free run procedure. Above that, Li-contamination is unlikely, since trace element data show an increase in all analysed elements. Furthermore, it cannot be excluded that the anomaly at 4.5 kbar is the only irregularity in the curve. As the irregularity covers a rather narrow range in pressure, further similar features could occur anywhere in between two existing data points.

**Fig. 9:**
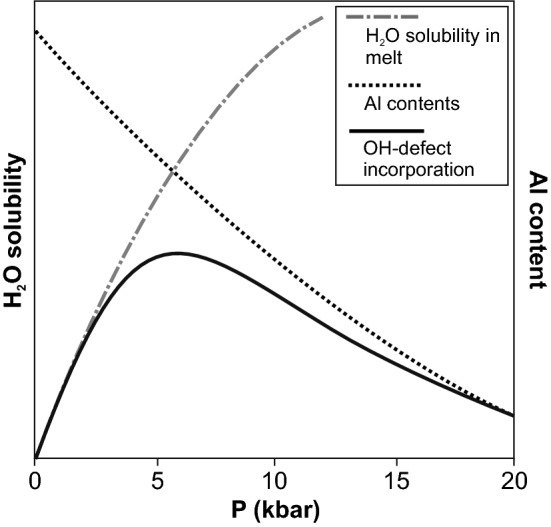
Schematic H_2_O solubility (arbitrary units) in melt (trend from Holtz et al. 1995) and Al content in quartz (Frigo et al. 2016). The combination of both curves (by multiplication) leads to a theoretical evolution of OH defect incorporation in quartz and justifies an OH defect maximum in the pressure range investigated in this study

If we consider the main factors for proton incorporation, a general picture emerges: Under the assumption that Al is always sufficiently present in granitic melts, OH-incorporation with pressure is controlled by the contrasting properties (1) solubility of water in the melt and (2) the compatibility of Al in the crystal lattice. Water solubility in silicate melts at 1 atm (and thus the OH incorporation into quartz grown from such a melt) is insignificantly low. Water solubility then increases with pressure, most dominantly between 1 atm and 1 kbar (Behrens 1995; Holtz et al. 1995), thus enhancing OH defect incorporation in quartz. This trend is not linear but decreases towards higher pressures. Al incorporation in quartz exhibits the opposite trend. Crystal/melt partition coefficients ($D_{{{\text{Quartz}}/{\text{melt}}}}^{{{\text{M}}^{{{\text{n}} + }} }}$) are a function of the lattice site radius and elasticity (Blundy and Wood 1994), leading to a decrease in incorporation of the larger Al^3+^ for Si^4+^ in quartz (and a lower $D_{{{\text{Quartz}}/{\text{melt}}}}^{{{\text{Al}}^{3 + } }}$) with pressure. This idea is supported by experimentally determined partition coefficients in the pressure range 7.5–20 kbar, where $D_{{{\text{Quartz}}/{\text{melt}}}}^{{{\text{Al}}^{3 + } }}$ decreases by a factor of 3 (Frigo et al. 2016). The combination of both factors with opposing pressure trends leads to a local maximum in OH defect incorporation (Fig. 9), which covers more or less the pressure range of this study (1–5 kbar), and explains lower OH content towards higher pressure (Frigo et al. 2016). The decrease of OH defects towards lower pressures (< 1 kbar) is predicted.

### Petrological implications and outlook

The variation of total OH defect incorporation between 1 and 5 kbar is too low to permit precise determination of the crystallisation depth. Nevertheless, the LiOH defect shows a promising narrow window of high contents between 4 and 5 kbar (Figs. 4 and 8) and is absent above 10 kbar (Frigo et al. 2016). Thus, it can be used as petrological indicator for crystallisation depths in systems containing elevated amounts of Li, particularly in late-stage granitic to pegmatitic systems. One important caveat is the fact that the LiOH maximum is observed in a narrow range and it cannot be excluded that a similar erratic behaviour can occur in between two pressures investigated here (e.g., 2.5 kbar). A narrower grid of experiments in the low-pressure regime (< 5 kbar) is necessary for reliable applicability as petrological tool. In addition, further experiments at low pressure (e.g., 0.2 and 0.5 kbar) should be performed to confirm the predicted rapid increase from 1 bar to 1 kbar. Results from these experiments would also be applicable to quartz bearing volcanic systems (Biro et al. 2017; Tollan et al. 2019).

## Conclusions

1. Experiments at 1–5 kbar on a natural granitic system do not show a clear trend in total OH defect content in quartz, and the average defect water content is around 200 µg/g. Between 4 and 5 kbar a sharp peak in OH incorporation up to 500 µg/g is observed, mainly caused by the increase in LiOH. The observed behaviour contrasts the negative OH incorporation trend to higher pressures (Baron et al. 2015; Frigo et al. 2016).
2. Trace element contents measured by SIMS analyses confirm the suggested charge balancing equation H^+^ + A^+^ + P^5+^ = M^3+^ + B^3+^ (Müller and Koch-Müller 2009).
3. The behaviour of OH defect incorporation with pressure is best explained by the opposing factors: (a) the water solubility in silicate melts increases with pressure, especially at lowest pressures; (b) the compression of the quartz crystal lattice inhibits the incorporation of Al (and the charge balancing protons) and thus reduces the crystal/melt partition coefficient.
4. In Li-bearing natural systems, quartz can be used as a rough petrological indicator for crystallisation depth up to 10 kbar, with pronounced LiOH defects at 4–5 kbar and rapidly decreasing LiOH towards 10 kbar.
